# M. Bérard

**Published:** 1859-03

**Authors:** 


M. BÉRARD, Professor of Physiology at the Faculty of Medicine of Paris, has recently died of apoplexy. On account of illness, he had not been able to lecture for three years, although he pursued his physiological investigations.

